# HIV Testing Implementation in Two Urban Cities: Practice, Policy, and Perceived Barriers

**DOI:** 10.1371/journal.pone.0110010

**Published:** 2014-10-13

**Authors:** Camden J. Hallmark, Jennifer Skillicorn, Thomas P. Giordano, Jessica A. Davila, Marlene McNeese, Nestor Rocha, Avemaria Smith, Stacey Cooper, Amanda D. Castel

**Affiliations:** 1 Houston Department of Health and Human Services, Houston, Texas, United States of America; 2 Department of Epidemiology and Biostatistics, School of Public Health and Health Services, George Washington University, Washington, DC, United States of America; 3 Center for Innovations in Quality, Effectiveness and Safety, Michael E. DeBakey VA Medical Center, Houston, Texas, United States of America; 4 Department of Medicine, Baylor College of Medicine, Houston, Texas, United States of America; 5 HIV/AIDS, Hepatitis, STD, and TB Administration, District of Columbia Department of Health, Washington, DC, United States of America; Rutgers University, United States of America

## Abstract

**Background:**

Although funding has supported the scale up of routine, opt-out HIV testing in the US, variance in implementation mechanisms and barriers in high-burden jurisdictions remains unknown.

**Methods:**

We conducted a survey of health care organizations in Washington, DC and Houston/Harris County to determine number of HIV tests completed in 2011, policy and practices associated with HIV testing, funding mechanisms, and reported barriers to testing in each jurisdiction and to compare results between jurisdictions.

**Results:**

In 2012, 43 Houston and 35 DC HIV-testing organizations participated in the survey. Participants represented 85% of Department of Health-supported testers in DC and 90% of Department of Health-supported testers in Houston. The median number of tests per organization was 568 in DC and 1045 in Houston. Approximately 50% of organizations in both DC and Houston exclusively used opt-in consent and most conducted both pre- and post-test counseling with HIV testing (80% of organizations in DC, 70% in Houston). While the most frequent source of funding in DC was the Department of Health, Houston organizations primarily billed the patient or third-party payers. Barriers to testing most often reported were lack of funding, followed by patient discomfort/refusal with more barriers reported in DC.

**Conclusions:**

Given unique policies, resources and programmatic contexts, DC and Houston have taken different approaches to support routine testing. Many organizations in both cities reported opt-in consent approaches and pre-test counseling, suggesting 2006 national HIV testing recommendations are not being followed consistently. Addressing the barriers to testing identified in each jurisdiction may improve expansion of testing.

## Introduction

HIV testing is an important step of the HIV care continuum and a critical component of prevention programs throughout the United States (US). Nationally, an estimated 18% of infected persons remain undiagnosed. [1] Awareness of HIV infection decreases risk-taking behaviors, [2] with earlier initiation of care resulting in decreased HIV-associated morbidity and mortality. [3], [4] In 2006, the US Centers for Disease Control and Prevention (CDC) released revised recommendations for routine, opt-out HIV screening in healthcare settings. Specifically, the revised recommendations include screening patients for HIV using an opt-out approach, the elimination of a separate written consent for HIV testing, and optional pre-testing prevention counseling. [5]

CDC funding has since supported the scale up of routine, opt-out screening nationwide, [6] yet implementation varies widely by jurisdiction as each determines how to expand testing given diverse contexts. Initiatives to scale up testing have included social marketing campaigns in Miami, FL and Oakland, CA, door-to-door testing outreach in San Diego, CA and Philadelphia, PA, [7] and policy change to mandate the offer of HIV testing in primary care settings in New York State. [8] Some models, such as that of the Bronx Knows HIV Testing Initiative, have demonstrated success through combination approaches in partnership with the local community and clinical providers. [7], [9]

This article reports on some of the similarities and differences in HIV testing observed in two jurisdictions highly impacted by HIV, Washington DC and Houston, TX. The jurisdictions are two of the twelve urban areas that represent 44% of the nation's AIDS cases. [10] DC has a generalized epidemic with 14,465 living HIV/AIDS cases and a prevalence of 2.7%, and Houston has 19,943 living HIV/AIDS cases and a concentrated epidemic with a prevalence of 0.6%. [11]–[13] Each jurisdiction's local epidemic and the associated policies, programmatic priorities, and resources that influence testing implementation are reported in Table 1.

**Table 1:** HIV Prevalence, Testing Policies and Programs by City.

	Washington, DC	Houston, TX^a^
***Local Data***		
Population (persons ≥13 yrs; Census estimates for 2010)	528,109	3,249,542
Number of living HIV/AIDS Cases ≥13 yrs (reported as of 2010)	14,465	19,943
HIV/AIDS Prevalence Rate (as of 2010)	2.7	0.6
Type of epidemic	Generalized	Concentrated
***Testing Guidance & Policies***		
Initiation of support for routine testing (year)	2006	2008
HIV testing consent and counseling regulations or statutes	No	Chapter 81 of Texas Health and Safety Code^b^
Mandatory HIV testing	Mandatory testing of convicted sex offenders within the criminal justice system	Mandatory testing of all offenders upon entry and release within Texas Department of Criminal Justice Texas law allows for mandatory testing in county and municipal jails
		Texas law allows for mandatory testing in county and municipal jails
Established HIV testing reimbursement and billing laws or regulations (year)	Insurance Coverage for HIV Testing in Emergency Departments Amendment Act (2008)	No specific laws on reimbursement or billing
***Programming & Resources***		
Estimated number of annual publically-funded HIV tests conducted	129,464 (2011)	107,458 (2011)
Testing campaigns/Programmatic initiatives/Research activities to scale-up HIV testing	Come Together DC Get Screened for HIV (2006)	HIP HOP for HIV Awareness (2007)
	Ask for the Test/Offer the Test (2009)	Expanded Testing Initiative (2008)
	Enhanced Comprehensive HIV Prevention Planning Initiative (2010)	Routine Screening for HIV- Memorial Hermann Health System and Project RUSH at Harris Health System (2008)
	HIV Prevention Trials Network 065 (intervention community) (2010)	Enhanced Comprehensive HIV Prevention Planning Initiative (2010)
		HIV Prevention Trials Network 065 (control community) (2010)

a Jurisdiction is Houston/Harris County. Population data are for Harris County.

b Since 1999, opt-out HIV testing has been required by Texas law during pregnancy at the first prenatal visit and at delivery. As of 2010, opt-out HIV testing is required during the third trimester. If no record of third trimester testing is found, opt-out testing at time of delivery is required. For all other persons (not incarcerated), general written consent for HIV testing is allowable. Informed consent may be verbal as long as test explanation and consent is documented in patient's medical record. Opt-in versus opt-out approach is not specified.

Policy in both jurisdictions has been relatively permissive of the expansion of routine HIV testing. Statutes relevant to the 2006 recommendations in DC and Texas were either neutral or consistent with all routine, opt-out recommendations. [14] Specifically, in DC, there are no regulations requiring written consent or pre/post-test counseling. The Insurance Coverage for HIV Testing in Emergency Departments (ED) Amendment Act, which was passed in 2008, attempts to facilitate third party coverage of testing in DC EDs. Under Texas Health and Safety Code, informed consent for HIV testing may be verbal as long as test explanation and consent are documented in a patient's medical record. Additionally, an opt-out approach is required by statute for pregnant women, where HIV screening is required at the first visit and during the third trimester. No statute requires pre-test prevention counseling and post-test counseling is only required upon delivery of a positive test result. [15]–[17]

Since 2006, the DC Department of Health (DOH) has supported targeted testing and routine, opt-out HIV testing implementation as a standard of care in health care settings. [18], [19] The DOH began this initiative by providing free rapid test kits and funding to testing programs at community-based organizations (CBOs), clinics, DC Department of Corrections, hospitals, and EDs. Between 2007 and 2008, DC expanded testing to additional hospitals, primary medical settings, managed care organizations, and CBOs. [20] The “Ask for the Test/Offer the Test” initiative launched in 2009 to educate providers on how to establish routine HIV testing protocols that emphasize the use of standard blood panels. The DOH also engaged the DC Department of Health Care Finance on promoting standard HIV testing as part of the DC Medicaid and Alliance programs. In 2010, DC DOH received funding from NIH as an intervention community for the HIV Prevention Trials Network (HPTN) Study 065 to support enhanced routine HIV testing and assess the feasibility of a test and treat strategy. [21] DC's DOH funded over 129,000 HIV tests in 2011, including the provision of over 122,000 rapid test kits.

In Houston, the CDC-funded Expanded Testing Initiative (ETI) started in 2008 when the Houston Department of Health and Human Services (HDHHS) began supporting routine, opt-out HIV testing in the EDs of two large hospitals and two community health centers. The Initiative grew to encompass four large EDs by 2009. With funding from the Texas Department of State Health Services (DSHS), routine testing was further expanded in 2010 to five additional EDs and twelve additional community health centers. Targeted testing was also funded by the HDHHS at CBOs for outreach to high risk populations and geographic areas. In 2011, the HDHHS funded over 107,000 tests in the Houston area.

Both DC and Houston health departments have provided support for the provision and funding of HIV testing in community and health care settings, including implementation of social marketing campaigns. HIV testing and promotion of HIV testing was further scaled up with the influx of HIV prevention activities funded by the CDC's Enhanced Comprehensive HIV Prevention Planning (ECHPP) Project in both DC and Houston. ECHPP was a 3-year demonstration project designed to improve program planning and implementation in support of the National HIV/AIDS Strategy. [22]

As part of ECHPP efforts, partnerships were formed between academic institutions and public health departments to improve program planning of HIV prevention activities in both DC and Houston. These partnerships ensured that local research capacity was improved and policy and programming stakeholders were invested in the study's outcomes, both of which are enabling factors for success in operational research. [23] While operational research has been utilized in low-income countries, [23], [24] it has been suggested that US jurisdictions could greatly benefit from applying an operational research approach to identify barriers and inform policy and decision-making in HIV prevention programs. [25] Therefore, in response to ECHPP's goal of improving implementation, we used descriptive operational research to gain knowledge of HIV testing implementation and organizational-level testing barriers in Washington, DC and Houston, Texas. Although implementation and barriers have been assessed among EDs, health centers, or clinics, [26]–[28] assessment across different types of organizations and jurisdictions has been limited. This study examined implementation mechanisms, testing volume, and organizational barriers for two cities with a high HIV/AIDS burden and provides a comprehensive jurisdictional-level perspective on HIV testing.

## Methods

### Survey instrument

Surveys were designed to gather organizational-level data on sites conducting HIV testing in each jurisdiction in 2011. Selected survey questions were adapted from the National Association of Community Health Centers survey on HIV testing, [29] a method used previously in similar research. [30] In both DC and Houston, organizations were questioned regarding their policies, funding, implementation practices, testing volume, and barriers to testing for calendar year 2011. Testing volume in 2011 was assessed by each organization providing counts by test type. Review of administrative and/or clinical data was the primary methodology used for gathering this data (80.0% of organizations in DC and 81.4% of organizations in Houston), followed by estimates from organizational staff members (17.1% DC and 11.6% Houston). Barriers to HIV testing were assessed by asking respondents to indicate the extent to which they agreed with possible barriers using a 5-point scale ranging from no barrier (1) to major barrier (5). Possible barriers included attitudinal conflicts, staff capacity, and available resources and funding.

### Sampling Frame

Sampling frames were designed to best fit the model of HIV prevention activities unique to each jurisdiction. In DC, the sampling strategy was developed through a partnership between DC DOH and researchers at the George Washington University as part of ECHPP. In 2011, DC DOH's HIV/AIDS, Hepatitis, STD, and TB Administration (HAHSTA)'s prevention model primarily supported HIV testing through distribution of HIV rapid test kits at no cost to approximately forty organizations. With stakeholder and DOH input, it was determined that these organizations conducted the vast majority of testing within the city. Therefore, organizations were identified for survey inclusion if their HIV testing services were HAHSTA-supported. These organizations included CBOs, clinics, and hospitals.

In Houston, the sampling frame and sampling tool were designed jointly by staff and researchers from HDHHS, Houston Area HIV Services Ryan White Planning Council's Office of Support, and Baylor-UTHouston Center for AIDS Research, also as part of ECHPP. The HDHHS funded two distinct HIV testing services at 10 organizations in 2011: 1) targeted HIV testing by CBOs that included prevention counseling, and 2) routine, opt-out HIV testing in EDs and county clinics. HIV testing in Houston was supported by a diverse mix of both public and private support, thus sampled organizations included both those that were and were not financially supported by the HDHHS. Utilizing a methodology described previously, [31] an exhaustive list of known HIV testing organizations was created from stakeholder input. The list included CBOs, substance abuse treatment centers, homeless shelters, hospitals, clinics and universities. This list was then prioritized to focus on obtaining information from HDHHS HIV prevention contractors, major public and private hospitals and clinics, and known HIV testing organizations.

### Survey Administration

In DC, surveys were emailed to HIV testing coordinators at each organization. Confidential surveys were administered online using Research Electronic Data Capture (REDCap). If no response was received within one month of invitation, paper surveys were mailed in addition to follow up phone calls. Participants were compensated for survey completion.

In Houston, each organization was emailed a formal invitation letter, followed by phone calls if there was no response via email. Initial contact was with HIV program directors, nurse managers, and/or lab directors. Respondents were selected based on their authority to make HIV testing program decisions and/or their knowledge of HIV testing activities within the organization. When possible, surveys were completed by an in-person interview, otherwise a telephone interview was conducted. Participants were not compensated for survey completion.

### Data Analysis

In DC, data collection and storage were via REDCap. Data analysis was conducted using SAS 9.2 (SAS Institute, Cary, NC). In Houston, all data were entered and managed in Excel. Data analysis was conducted using SAS 9.3 (SAS Institute, Cary, NC). Summary data from both jurisdictions were compiled via Excel and analyzed collaboratively by researchers from both jurisdictions.

### Consent

This study was approved by the Institutional Review Boards of The George Washington University, the District of Columbia Department of Health (Washington, DC) and the Baylor College of Medicine (Houston). In Houston, the Institutional Review Board approved verbal consent, with completion of the survey as documentation of consent. Consent was also documented in a secure database by the interviewer. Written consent would have been the only record linking the participant and survey; therefore, the potential harm resulting from a breach of confidentiality would have increased since no other personal identifiers were gathered. In DC, the Institutional Review Board approved the use of written consent. The survey instructions asked participants to mark “yes” or “no” to indicate their consent to the survey prior to answering any further questions. In both jurisdictions, the survey was voluntary and deemed no more than minimal risk to participants.

## Results

### Respondents & Organizational Characteristics

In DC, 41 organizations were contacted for study inclusion. Of these, 35 (85.4%) organizations participated in the survey, representing 103 facilities that conducted HIV testing. Many of the DC organizations were community-based organizations/community service organizations (40.0%) (Table 2).

**Table 2:** HIV Testing Policy, Funding, and Implementation Practices.

	Washington, DC (N = 35)		Houston, TX (N = 43)	
	N	%	N	%
**Type of organization**				
Hospital	12	34.3%	11	25.6%
Community-based or community service organization^a^	14	40.0%	10	23.3%
Clinic or university health center	9	25.7%	22	51.2%
**Individual at organization who coordinates HIV testing efforts**				
Yes	29	82.9%	27	62.8%
No	6	17.1%	16	37.2%
**Have a written policy for HIV testing**				
Yes	29	82.9%	20	46.5%
No	6	17.1%	23	53.5%
**How providers are educated about HIV testing** c				
Continuing education	26	74.3%	20	46.5%
DOH or clinic training	23	65.7%	25	58.1%
Peer-to-peer best practices	20	57.1%	17	39.5%
Manuals, guidelines, or literature	16	45.7%	21	48.8%
Web seminars	12	34.3%	11	25.6%
Consulting site visits	6	17.1%	11	25.6%
In-person or video conferences	5	14.3%	10	23.3%
Other	1	2.9%	1	2.3%
None reported	2	5.7%	9	20.9%
**Type of consent procedure for HIV testing** b				
Opt in approach ONLY	18	51.4%	21	48.8%
Opt out approach ONLY	17	48.6%	3	7.0%
Combination of opt in and opt out	0	0.0%	19	44.2%
**Type of HIV counseling offered with testing**				
Pre-test counseling only	1	2.9%	1	2.3%
Post-test counseling only	4	11.4%	7	16.3%
Both pre and post-test counseling	28	80.0%	30	69.8%
Neither pre nor post-test counseling	1	2.9%	5	11.6%
Other	1	2.9%	0	0.0%
**Funding used by organization for HIV testing** c				
Local Health Department Funding (DC, Houston)	26	74.3%	10	23.3%
Texas DSHS	N/A	N/A	8	18.6%
CDC Funding	9	25.7%	3	7.0%
Other Federal Funding (Ryan White, Other HRSA, SAMHSA, VA)	11	31.4%	7	16.3%
Private Grant Funding	7	20.0%	8	18.6%
Research (HPTN 065/TLC Plus HIV testing or other research study)	12	34.3%	2	4.7%
Medicaid	10	28.6%	23	53.5%
Medicare	4	11.4%	14	32.6%
Patient/patient's insurance	10	28.6%	28	65.1%
Other d	1	2.9%	14	32.6%
None reported	12	34.3%	0	0.0%
**Funding used by organization for routine, opt out testing (among those doing opt out testing)** c	***n = 35***		***n = 22***	
Local Health Department Funding (DC, Houston)	24	68.6%	4	18.2%
Texas DSHS	N/A	N/A	5	22.7%
CDC Funding	5	14.3%	0	0.0%
Other Federal Funding (Ryan White, Other HRSA, SAMHSA, VA)	6	17.1%	1	4.5%
Private Grant Funding	5	14.3%	6	27.3%
Research (HPTN 065/TLC Plus HIV testing or other research study)	5	14.3%	0	0.0%
Medicaid	9	25.7%	11	50.0%
Medicare	4	11.4%	3	13.6%
Patient/patient's insurance	7	20.0%	12	54.5%
Other e	1	2.9%	9	40.9%
None reported	6	17.1%	0	0.0%
**HIV tests performed in 2011**				
	Median (Range)	Total No. of Tests	Median (Range)	Total No. of Tests
Rapid tests (DC n = 30; Houston n = 21)	593 (5–10,673)	78,765	1,478 (4–9,945)	40,910
Venipuncture tests (DC n = 3; Houston n = 34)	1200 (36–3,322)	4,558	585 (1.5–46,590)^f^	169,635
Total tests (DC n = 22; Houston n = 41)	568 (20–10,673)	41,085	1,045 (1.5–47,209)^f^	210,565

a Community service organizations include homeless services, substance abuse recovery centers, life skills programs, housing assistance programs, faith-based organizations, and other community-oriented service organizations.

b Five organizations participating in the Houston survey only use opt-out consent during pregnancy as required by Texas law but do not use opt-out consent otherwise. Therefore, the bulk of consent used for these organizations would be opt-in. Results if these organizations are re-classified into the “opt-in approach only” category: 26 (60.5%) organizations use opt-in approach only, 3 (7.0%) organizations use opt-out approach only, and 14 (32.6%) organizations use a combination of opt-in and opt-out consent.

c Respondents could check more than one response.

d Other responses in DC include general revenue (n = 1). Other responses in Houston include donations (n = 12) and general revenue (n = 2).

e Other responses in DC include general revenue (n = 1). Other responses in Houston include donations (n = 8) and general revenue (n = 2). One Houston organization selected both donations and general revenue for a total of n = 9 organizations selecting “other”.

f One Houston organization estimated 1–2 tests were completed. The average, 1.5 tests, was recorded as the response.

In Houston, 84 organizations were contacted for study inclusion. Of these, 55 (65.5%) organizations participated in the survey. Forty-three participating organizations, representing 114 facilities in the Houston/Harris County area, conducted HIV testing. This analysis focused only on the 43 organizations conducting HIV testing of which over half (51.2%) were outpatient clinics or university health centers (Table 2).

### Policy and Practices, Funding, and Testing Volume

The majority of organizations reported that a specific individual coordinated HIV testing efforts for the organization. DC organizations more frequently reported a coordinator than did Houston (82.9% and 62.8%, respectively) (Table 2). Similarly, 82.9% of surveyed organizations in DC reported having a written policy for HIV testing, while only 46.5% of surveyed organizations in Houston reported having a policy for their organization.

The two jurisdictions reported similar approaches to provider education about HIV testing. The top three sources of provider education for DC were continuing education (74.3%), DOH or clinic training (65.7%), and peer-to-peer best practices (57.1%). Houston organizations most frequently identified DOH or clinic training (58.1%), manuals/guidelines/literature (48.8%), and continuing education (46.5%).

A similar proportion of organizations exclusively used an opt-in consent approach in DC and Houston (51.4% and 48.8% respectively). However, a substantial number of organizations in Houston used a combination of opt-in and opt-out approaches (44.2%). Further analysis revealed that 11.6% (n = 5) of organizations in Houston using a combination approach only used opt-out consent during pregnancy. While only three (7.0%) organizations in Houston reported using only the opt-out approach, 48.6% of the surveyed organizations in DC exclusively used an opt-out approach.

Despite CDC recommendations that removed the need for pre-test counseling, most organizations in both jurisdictions reported conducting both pre- and post-test counseling with HIV testing (80.0% in DC and 69.8% in Houston). Very few organizations reported conducting only post-test counseling (11.4% in DC and 16.3% in Houston).

For all HIV testing conducted by surveyed organizations, the most frequent funding sources for DC were the DOH (74.3%), research monies (34.3%), and federal funding other than that from CDC and DOH (33.3%). In contrast, Houston organizations most frequently identified the payer as the patient or the patient's insurance (65.1%), Medicaid (53.5%), Medicare (32.6%), and other sources (32.6%) such as the organization's general revenue or funds received as donations. Funding specifically for routine, opt-out HIV testing also varied between the two cities. The most frequent funding source in DC was the DOH (68.6%), while Houston organizations primarily paid for HIV testing by billing the patient or patient's insurance (54.5%) or Medicaid (50.0%).

The median total number of HIV tests performed per organization in 2011 was higher in Houston than in DC (1045 and 568 respectively). While the total number of rapid tests performed in DC was higher than in Houston (78,765 in DC vs. 40,910 in Houston), the median number of rapid tests performed per organization was lower in DC than in Houston (593 vs. 1478). Similarly, although the median number of venipuncture tests per organization was higher in DC (1200 in DC vs. 585 in Houston), the total number of venipuncture tests was higher in Houston than in DC (169,635 in Houston vs. 4,558 in DC).

### Barriers to HIV Testing

**Figure 1:**
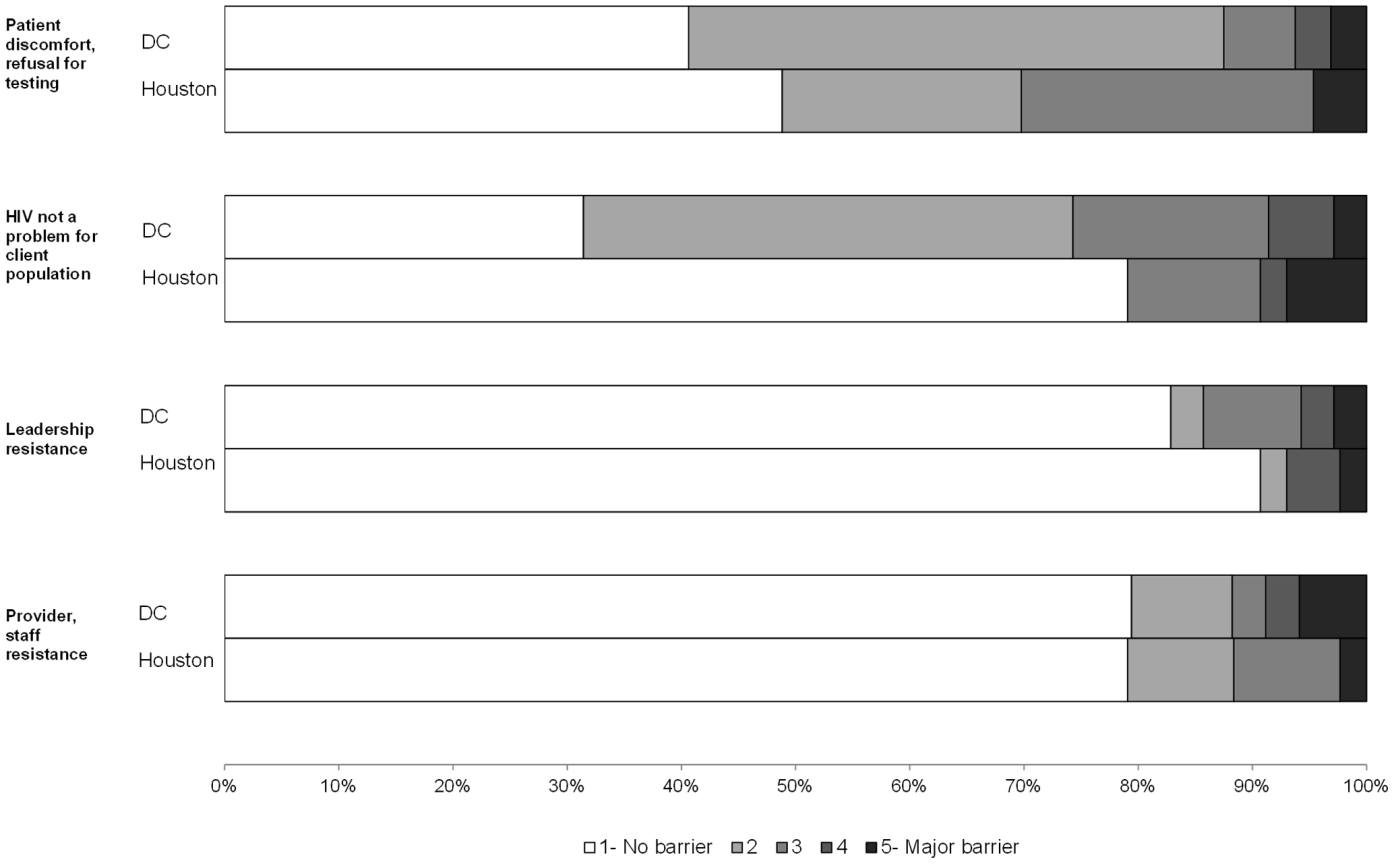
Attitudinal barriers to HIV testing by City.

**Figure 2:**
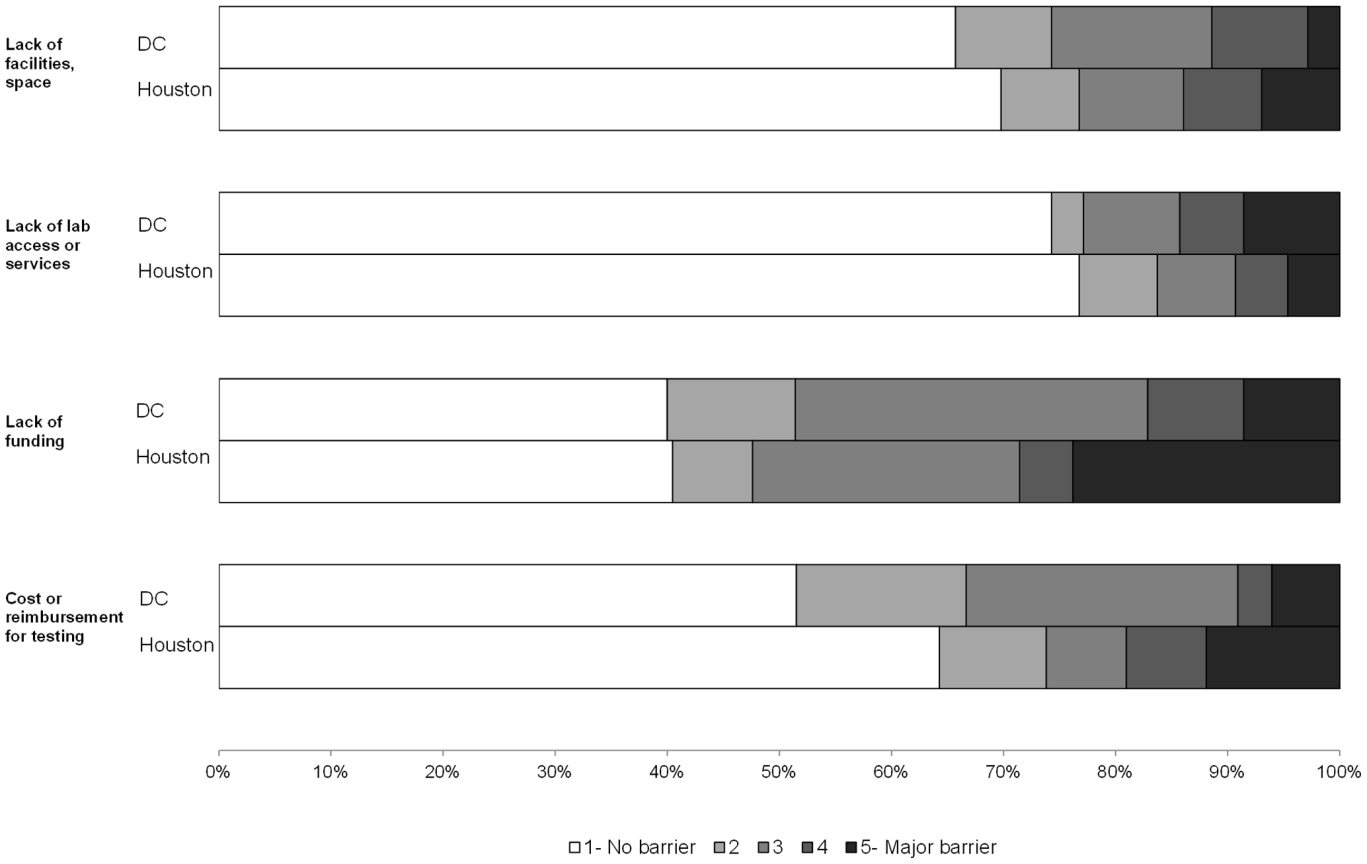
Resources and funding barriers to HIV testing by City.

**Figure 3:**
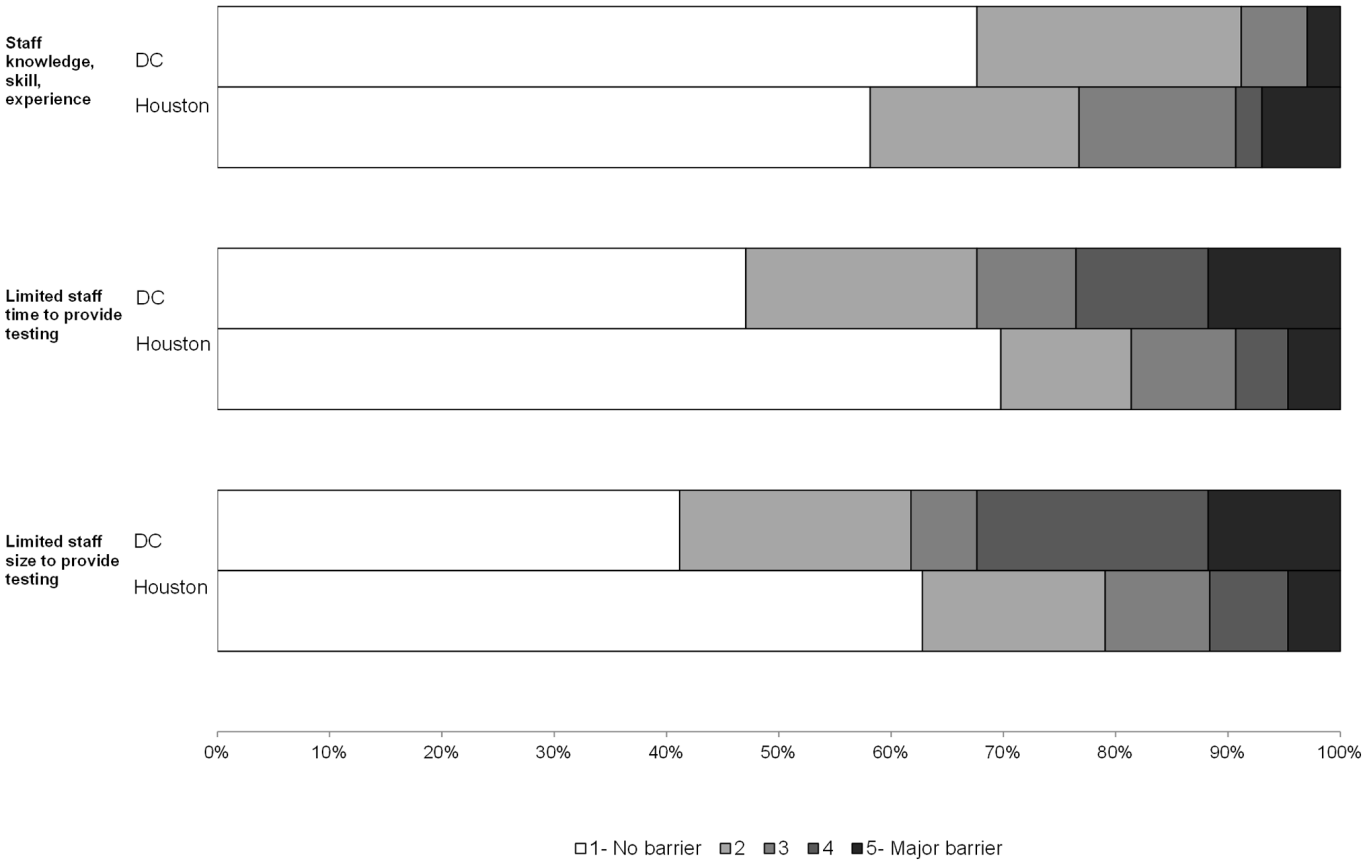
Staff capacity barriers to HIV testing by City.

On a scale of 1 (no barrier) to 5 (major barrier), the highest reported barriers to HIV testing in both DC and Houston were patient discomfort/refusal (median score  = 2) and lack of funding for testing (median score  = 3). When categorizing all items dichotomously (no barrier vs. any barrier), DC organizations most frequently selected “HIV not a problem for client population” (68.6% of organizations), “lack of funding” (60.0%), and “patient discomfort or refusal for testing” (59.4%) as barriers (Figures 1–3). Houston organizations most frequently selected “lack of funding” (59.5% of organizations), “patient discomfort or refusal for testing” (51.2%), and “staff knowledge, skill, experience” (41.9%).

In comparison to Houston organizations, DC organizations more frequently reported every area as a barrier except for staff knowledge, skill, experience (41.9% Houston vs. 32.4% DC) and provider/staff resistance (20.9% vs. 20.6%) (Figures 1–3). However, 11 Houston organizations (25.6%) scored “other” barriers to testing at 3 or higher, including managing data and/or paperwork associated with testing and funding for testing (n = 4) and challenges in contacting patients for results delivery and/or care referrals (n = 3). The largest variances observed between the two jurisdictions were “HIV is not a problem for the client population” (20.9% Houston vs. 68.6% DC), “limited staff time to provide testing” (30.2% vs. 52.9%), and “limited staff size to provide testing” (37.2% vs. 58.8%).

We examined whether barriers to testing implementation differed by organization type (CBOs, hospitals, and clinics; data not shown). Lack of funding was reported frequently across all organizational types. Clinics were particularly challenged by cost or reimbursement for testing and patient refusal, while CBOs reported staff size as a barrier. Among hospitals, the highest ranked barrier to testing in both jurisdictions was the perception that HIV was not a problem in their client population. In DC, barriers to testing were most frequently reported among hospitals in comparison to other site types. The most frequent barriers among DC hospitals included the perception that HIV is not a problem in the client population (83.3% of organizations), staff time (75.0%), staff size (75.0%), and lack of funding (66.7%). In contrast, in Houston, hospitals reported the fewest barriers, while clinics reported the most. The most frequent barriers among Houston clinics included lack of funding (68.2%), refusal to get tested (63.6%), cost/reimbursement (50.0%), and staff knowledge/skill/experience (50.0%).

## Discussion

Utilizing a framework of operational research for HIV prevention, [25] this study identified differences and similarities in the approaches two high prevalence cities have taken to implement routine HIV testing. While both jurisdictions reported a high level of provider education on HIV testing, many organizations in both cities reported opt-in consent approaches and pre-test counseling, suggesting the 2006 CDC recommendations are not being followed consistently. The cause of inconsistency merits further research since statutes do not pose a substantial barrier in either jurisdiction. [14] Previous implementation research has shown that this inconsistency may be due to 1) a lack of awareness or misunderstanding of the recommendations, [27], [32], [33] 2) disagreement with the recommendations, [34] and/or 3) organizational barriers that impede application of the recommendations. [27], [28], [30], [35], [36] Both cities may benefit from further research into why organizations continue to use both pre- and post-test counseling and do not use opt-out testing.

The two jurisdictions have taken different approaches to support and fund the expansion of routine testing. DC DOH's approach has involved considerable funding for the distribution of free rapid test kits to local implementation sites including clinics, hospitals and CBOs. This is in contrast to the HDHHHS' approach which has encouraged performing venipuncture tests as part of routine visits and funding large volumes of HIV testing in hospitals and clinics with rapid results of standard testing. [37] This divergence of approaches is reflected in the greater total number of rapid tests being utilized in DC and much greater total number of venipuncture tests being performed in Houston.

Funding sources for testing also varied between the two jurisdictions which could have implications for the long term sustainability and scalability of these testing programs. Houston testing organizations reported much more third party billing and reimbursement from Medicaid, Medicare, and private insurance as a primary means to support their testing programs, compared to DC sites which relied much more heavily on direct support from DC DOH. Because of this, Houston organizations may be better positioned to more quickly adapt and benefit from expanded coverage through the Affordable Care Act (ACA), especially now that routine testing is rated a recommended service for coverage by the US Public Health Services Task Force (USPHSTF). However, this strong reliance on third party reimbursement does leave these organizations more vulnerable to reimbursement challenges and inadequate reimbursement rates that may not cover the total cost of testing provision. The monitoring of HIV testing post-ACA and USPHSTF implementation will allow for further exploration of the impact of these policies on testing programs.

Overall, few barriers to testing implementation were reported by organizations in both jurisdictions however, among those reported, lack of funding and organizational capacity to conduct testing were key barriers. Capacity barriers, such as staff size and time were moderate barriers for the surveyed organizations and may have been related to lack of funding to support these efforts at the organizational level, particularly among organizations in DC. Although multiple methods of routine HIV testing have been promoted in DC, there is more reliance on rapid testing, which can be more time and labor-intensive than venipuncture testing. Time-related barriers and competing priorities have been extensively reported in literature, [26]–[28], [30], [32], [35], [38], [39] but research has found that providers cite consent and counseling requirements as time-intensive even when statutes have removed these barriers in a jurisdiction. [26] Interestingly, despite the generalized epidemic and high HIV prevalence in DC, a commonly reported barrier included the perception that HIV was not a problem among the patient population. This perception may depend on the age of the population being served as a previous survey conducted by DC DOH found that almost 40% of providers did not perceive HIV to be a problem among those 50 years of age and older [Michael Kharfen, personal communication]. Barriers elicited in this study suggest that DC may benefit from campaigns that emphasize the necessity of HIV testing, while Houston may benefit from increasing testing knowledge and skill among providers. Patient discomfort or refusal for testing was a barrier in both cities and has been reported by providers elsewhere [26], [27], [39] with clients often refusing due to low-perceived risk for HIV, recent testing elsewhere, [40], [41] fear of loss, fatalism, confidentiality concerns, and structural barriers. [36] Future qualitative research in both DC and Houston, focusing first on the commonly reported barriers presented here, may best elicit additional barriers and facilitators of sustained HIV testing.

This study determined current implementation practices and perceived barriers to HIV testing, a crucial step to design interventions and guide policy to improve HIV testing in cities with a high burden of HIV, such as DC and Houston. In 2010, New York State enacted a statute requiring primary care settings to offer HIV testing to all patients between the ages of 13 to 64. Since this law was enacted, one study found that only 65% of emergency departments implemented HIV testing as required by the law. [8] While changes to policy guidance at the jurisdictional level may increase testing, [42] statute alone is not the solution. [8], [35] Further increases in testing uptake may be realized with policy change and implementation at the organizational level, [43], [44] especially in jurisdictions with low uptake such as some organizations in Houston. Organizational leadership to drive local policy formulation, revision, and implementation is critical. Other facilitators include organizational buy-in from both providers and administration, [27], [28] written procedures for HIV testing, [30] provider trainings, [27] and dedicated staff and funding. [35]

There are a number of limitations to this study. The survey was a convenience sample that could be subject to non-response bias. The self-reported responses were limited to the organizational knowledge, perceptions and/or experiences of the person completing the survey, thus the responses may not accurately reflect the practices or opinions of all providers or staff members. The primary limitation of the parts of this study that compared DC and Houston was differences in survey administration between the two cities. Non-response was potentially influenced in Houston (65.5% vs. 85.4% in DC) by the lack of compensation for survey completion. The differing routes of survey administration (in-person in Houston and online in DC) may have also led to variances in social desirability or comprehension of the survey questions. In addition, social desirability may have biased the results, especially in reporting of testing barriers. Furthermore, our ability to statistically analyze barrier data was limited as many of the barriers received low rankings. Finally, generalizability of these findings may be limited, particularly in jurisdictions that have statutes in conflict with the 2006 CDC recommendations. Results presented herein may be useful to such jurisdictions after their statutes transition.

Despite both DC and Houston having high HIV prevalence, each jurisdiction has tailored their HIV testing strategies to meet particular funding options, resources, and statutes. The range of approaches was appropriate given the distinct context of the epidemic in each jurisdiction. Reported implementation practices, such as types of tests used and the funding mechanisms most often used to pay for HIV testing, highlight this variation. Regardless of different implementation approaches, our results suggest many organizations in at least two high burden jurisdictions have not aligned with all aspects of national testing recommendations. Research has shown that neither awareness of CDC guidelines nor jurisdictional policy change alone fully scales up testing to the levels recommended in the 2006 guidelines. [8], [32], [39] It is likely that a multi-faceted approach is needed that includes awareness campaigns, policy change, and reduction of jurisdictional and organizational-specific barriers. Few multi-faceted approaches have been rigorously evaluated to determine if they can widely span multiple jurisdictions with diverse epidemics, policy, and resources. Further implementation research and dissemination of implementation research findings is critical to the expansion of routine HIV testing and early diagnosis of HIV infection.
